# Physical work environment in an activity-based flex office: a longitudinal case study

**DOI:** 10.1007/s00420-024-02073-z

**Published:** 2024-05-17

**Authors:** Viktoria Wahlström, Maria Öhrn, Mette Harder, Therese Eskilsson, Anncristine Fjellman-Wiklund, Anita Pettersson-Strömbäck

**Affiliations:** 1 Department of Public Health and Clinical Medicine, Umeå University, Umeå, Sweden; 2 Umeå School of Architecture, Umeå University, Umeå, Sweden; 3 Department of Community Medicine and Rehabilitation, Physiotherapy, Umeå University, Umeå, Sweden; 4 Department of Psychology, Umeå University, Umeå, Sweden; 5 Section of Sustainable Health, Department of Public Health and Clinical Medicine, Umeå University, Umeå, 901 87 Sweden

**Keywords:** Ergonomics, Flexible office, Office design, Occupational health and safety, Office workers, Work environment

## Abstract

**Objective:**

This study aimed to investigate and explore Occupational Health and Safety (OHS) management, office ergonomics, and musculoskeletal symptoms in a group of office workers relocating from cell offices to activity-based flex offices (AFOs).

**Methods:**

The analysis was based on qualitative interview data with 77 employees and longitudinal questionnaire data from 152 employees.

**Results:**

Results indicate that there was a need to clarify roles and processes related to the management of OHS. Self-rated sit comfort, working posture, and availability of daylight deteriorated and symptoms in neck and shoulders increased after the relocation and seemed to be influenced by many factors, such as difficulties adjusting the workstations, the availability of suitable workplaces, and age, sex, and individual needs.

**Conclusion.:**

Research on the long-term effects of physical work environments and management of (OHS) issues after implementing activity-based flex offices is sparse. This study demonstrates the importance of planning and organising OHS issue management when implementing an AFO, and to carefully implement office ergonomics among office workers.

**Supplementary Information:**

The online version contains supplementary material available at 10.1007/s00420-024-02073-z.

## Practitioner summary

Knowledge of effects on the physical work environment and management of Occupational Health and Safety (OHS) issues after implementing activity-based flex offices (AFOs) is sparse. Findings from this mixed methods longitudinal study suggests a possible need to reorganise the management of OHS issues and carefully implement office ergonomics when implementing flexible office types like AFOs.

**Supplementary Information:**

The online version contains supplementary material available at 10.1007/s00420-024-02073-z.

## Introduction

In recent years there has been increased interest in flexible offices. Technological developments enable office workers to work from different locations, and it is more common to transfer to Flex Offices with Activity-based work, abbreviated as ‘AFOs’ (Rolfö 2018; Engelen et al. 2019). The main reasons for implementing AFOs are usually to stimulate communication and collaboration, and to decrease facility costs (Engelen et al. 2019; Haapakangas et al. 2019). In AFOs there are no individually assigned workstations. Instead, the choice of space is based on the current activity in terms of work task, and workers can choose a suitable workstation based on the task at hand and personal preferences. AFOs are usually designed to provide support for different types of work tasks, such as spaces for individual concentrated work, spaces for meetings and social areas, and under optimal conditions, there is always access to the right type of workstation (Hoendervanger et al. 2018).

The intention of Occupational Health and Safety (OHS) in the workplace is to maintain and promote worker health and capacity, and to develop and improve the occupational setting. This is preferably performed through a process that includes a continuous loop of evaluations, planning, and the implementation of improvements (ILO 2001). OHS issue management likely varies depending on the country and organisation, as it is often guided by legislation, company policies and routines. When transferring to flexible offices, like AFOs, there are likely to be changes in the way OHS issues are handled if the AFO design leads to sharing facilities across departments within the organisation.

Musculoskeletal symptoms are common among office workers, prevalence is higher in females than in males (Lucas et al. 2022; Janwantanakul et al. 2008), and the prevalence of musculoskeletal symptoms also increase with age (Hoy et al. 2010; Mills et al. 2019). Preventive measures are important in avoiding the development of musculoskeletal symptoms (Hoe et al. 2018). Relocation to AFOs could positively affect workers due to increased variation, but it may also pose an ergonomic risk, as workers do not have individually adjusted workstations. In general, interventions to increase standing and walking among office workers have not been shown to reduce musculoskeletal symptoms (Parry et al. 2019), studies performed in AFOs have demonstrated a reluctance toward workplace-switching in AFOs (Hoendervanger et al. 2016; Haapakangas et al. 2018), and studies using objective measurements have seen only limited effects on occupational sitting patterns (Wahlström et al. 2019; Johansson et al. 2020). Another important ergonomic aspect of importance in office environments are visual conditions, including the lighting system, the position of computer monitors, and the visual acuity of the worker (Robertson et al. 2016). Under optimal conditions, workers are capable of performing tasks without straining the eyes (Osterhaus et al. 2015). Several visual changes can occur during human ageing, such as presbyopia, decreased contrast sensitivity, and delayed glare recovery (Peiyi 2014; Erdinest et al. 2021), therefore visual ergonomics at the workplace might affect younger and older workers differently.

Meijer et al. (2009) studied office workers who were relocated to an innovative office concept and found a decreased prevalence of upper extremity complaints at follow-up 15 months after relocation. In a qualitative study, Babapour et al. (2019) investigated consequences of desk-sharing in four organisations after relocation to AFOs and found that employees perceived that they sat less and moved more, but also noted inconveniences related to setting up and changing workstations. Berthelsen et al. (2018) found decreased ratings for workplace design regarding sit comfort and work posture among Swedish university employees after relocation to an AFO. When it comes to visual conditions for computer work in flexible office, to our knowledge, there are no studies reported in the current literature.

To achieve workplace prerequisites for good health and high productivity, it is important to establish ways of organising and managing OHS to provide workers with sustainable working environments in flexible offices. This has become even more important in the post-pandemic situation, which has trended towards a hybrid organisation of office work; i.e., remote work from home combined with work in flexible offices (Chafi et al. 2021; Chan et al. 2022). Remote work is likely to impact ergonomics and OHS management, and responsibility for the work environment may become even more complex compared to working in an AFO. To date there is limited and mixed evidence on the effects of the physical work environment, office ergonomics, and musculoskeletal disorders after relocation to AFOs, and more studies are needed (Lahtinen et al. 2015; Engelen et al. 2019; Haapakangas et al. 2023).

In a previous study, Öhrn et al. (2021), results showed that employees relocating from cell offices to an AFO reported reduced sit comfort and work posture after relocation, when compared to a control group that kept working in cell-based offices. To achieve a deeper understanding of office ergonomics and the management of OHS in AFOs, the overall aim of this study was to investigate and further explore the physical work environment in a group of office workers who relocated from a cell-based office to an activity-based flex office using mixed methods. The research questions were:

- What are the important experiences for OHS management in an activity-based flex office (AFO)?
- How are office ergonomics and musculoskeletal symptoms affected after relocation, and what seems to influence outcomes?

## Materials and methods

### Setting

The current study is part of an overarching research project, the Active Office Design Study (AOD), a longitudinal quasi-experimental office relocation project (Wahlström et al. 2019, 2020; Öhrn et al. 2021). Before relocation, 91% of workers had a personal workstation and 71% worked in personal cell offices, and they relocated to either a new cell office or an AFO. In the current study, we included white-collar workers who relocated to the AFO, including workers in finance, human resources, urban planning, educational departments, and a group of politicians. In the studied organisation, managers had a delegated responsibility for the work environment of their employees.

Before the relocation managers regularly followed up on the physical work environment by, e.g., performing yearly safety inspections in the areas/corridors used by their employees. Before relocation, most employees had individual workplaces, adjustable chairs, and sit–stand tables. Ergonomists from the off-site occupational healthcare provider screened the workstation ergonomics every second year. Safety officers were assigned to represent co-workers in different departments, and to take part in developing the work environment, together with managers. Managers were mandated to implement improvements in the work environment. As part of the AOD study, an intervention program aiming to decrease sitting, increase standing and walking, and to break up prolonged sitting periods at work was developed and implemented. (Wahlström et al. 2019, 2020).

After the relocation, the AFO had three floors containing open office landscapes, secluded offices, and conference rooms of various sizes. There were no assigned zones for different types of silent or more interactive work activities. The secluded offices were thought to be used whenever needed, depending on work tasks and various individual needs. After the relocation, employees had non-assigned workstations. The workstations had a standardised design, with adjustable chairs of the same model, sit–stand tables, and adjustable computer monitors. Some meeting rooms had sit–stand tables. Both sitting and standing tables were available in shared spaces. In addition, 16 shared treadmill workstations were installed in both cell offices and open office landscapes. More details are found in Supplementary Table 1.

When planning the office design, the organisation consulted experts in workplace design, as well as ergonomists from the occupational health services. After relocation, employees were invited to join two non-mandatory ergonomic training sessions at the workplace, performed by ergonomists. After the demonstration, employees were offered a personal ergonomic consultation if they wanted them. During the planning phase, the organisation performed regular risk assessments in collaboration with union representatives.

### Study design

**Fig. 1:**
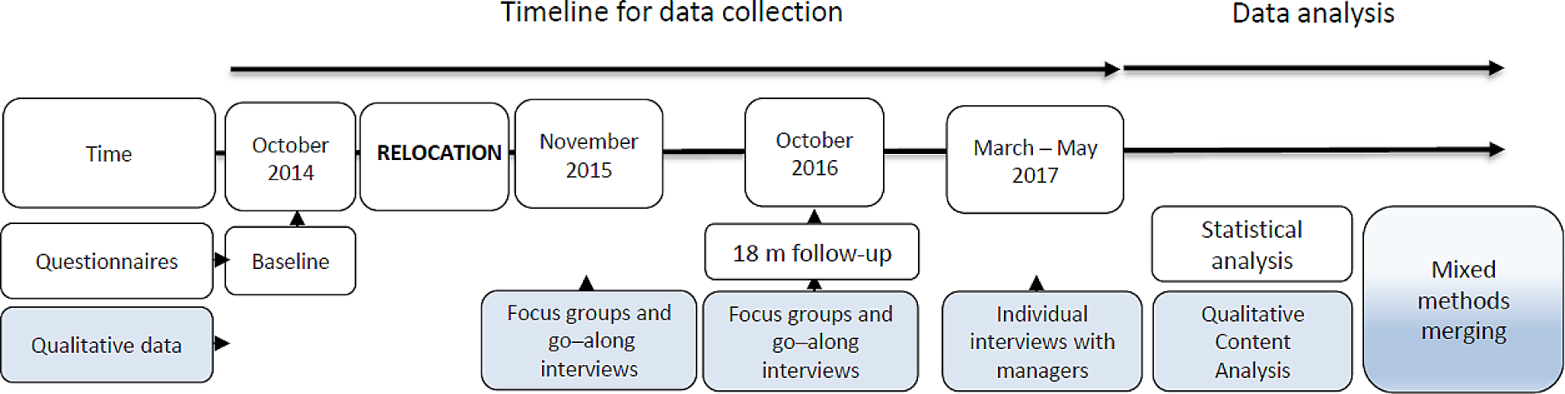
Flow chart for data collection and data analysis in the mixed methods design.

We used a mixed methods design integrating qualitative and quantitative data (O’Cathain et al. 2008; Fetters et al. 2013). The use of mixed methods provides the possibility of a deeper understanding of the research questions, as the qualitative and quantitative datasets complement each other (Fetters et al. 2013; Morse and Cheek 2014). In the qualitative part, interviews were undertaken to explore employees’ experiences from the relocation process and the physical work environment. In the quantitative part, questionnaires were used to assess background characteristics, health, and perceived work environment. This approach provided the possibility to investigate perceptions of the physical ergonomic environment and any related behavioural changes. The study had a convergent design, meaning that qualitative and quantitative data were collected in parallel but separately, and analysis and data integration were carried out after data collection was completed (Fetters et al. 2013) (Fig. 1). We present the results using an exploratory sequential mixed methods design, where the qualitative results are considered the main findings, and the quantitative findings are used to give an additional dimension to the results, presented in a weaving pattern (Fetters et al. 2013; Fetters and Freshwater 2015).

The AOD study received ethical approval from the Regional Ethical Committee (No:2014/226 − 31). All participants signed an informed consent form with separate information and consent forms for the collection of questionnaires and interview data in the study. Data were collected between October 2014 and May 2017.

### Data collection and analysis

#### Qualitative data collection with interviews

Qualitative data were collected through interviews, with a total of 13 focus groups, 6 individual interviews with managers, and 7 go-along interviews (Gill et al.^2008^; Carpiano 2009). Convenience sampling was used for recruitment, where all employees relocating to the AFO were invited to participate. Go-along interviews were conducted six months after relocation and focus groups at 6 and 18 months after relocation. One of the focus groups (at six months) only included managers. Due to challenges scheduling a focus group with the managers at 18 months, individual interviews with managers were carried out 24 months after the relocation (Fig. 1).

A semi-structured interview guide with open-ended questions covering experiences from the relocation process to specific questions about the physical work environment, ergonomics, and musculoskeletal disorders was used for all interviews (Gill et al.^2008^) Questions about the organisation and management of OHS were included in the focus groups at 18 months and in the manager interviews. Focus groups lasted 50–90 min, involved 3–5 participants per group, and were held by two researchers. Manager interviews were also held by two researchers and lasted 30–60 min. Go-along interviews are an architectural method to capture participants’ perceptions of the built environment (Carpiano 2009). Each go-along interview lasted 90 min, was held by one of the researchers, and involved 2–6 participants per group. Guided by a researcher, each group visited the same six predetermined places in the office. At each place, the participants were asked to reflect on the positive and negative aspects of the space and on how well-suited it was for sitting, standing, or walking. The participants were also encouraged to suggest changes that could improve the place. The participants noted down their reflections on an architectural drawing of the office. Participants were allowed to discuss these places during the walk and, when this happened, were reminded by the researcher to note down the discussions. After walking around, the group gathered for an interview in a meeting room. Participants summarised their reflections, and a joint discussion about the work environment and ergonomics was held. The interview was recorded, and the participants’ notes were collected. All interviews were recorded and transcribed verbatim.

#### Qualitative content analysis

Interview data were analysed using qualitative content analysis (QCA) with an inductive approach (Graneheim and Lundman 2004; Elo et al. 2014; Graneheim et al. 2017). Before analysis, the first author extracted data relevant to the aim of the study from the full interviews. Three of the authors began by reading the selected transcribed excerpts. To validate the coding procedure, they collectively extracted condensed meaning units and abstracted codes from one focus group. In the next step, each researcher coded an information-dense interview. The last step was a mutual comparison and a final negotiated outcome. Data were then divided among the authors, who individually extracted codes. The resulting codes were then presented to all authors. Three of the authors analysed the data, grouping them into preliminary themes, categories, and subcategories. To further strengthen trustworthiness, preliminary results were presented and discussed with all authors on four occasions, and with other researchers involved in the AOD study on two occasions (Graneheim and Lundman 2004; Elo and Kyngäs 2008; Graneheim et al. 2017). Repeated reflections and feedback on the preliminary results guided further discussions and any modifications made to the analysis.

#### Quantitative data collection with questionnaires

All employees involved in the relocation were invited to a questionnaire 6 months before the relocation (baseline), and at 6 and 18 months after the relocation. Questionnaires were distributed via managers, with a sealable return envelope, and could be answered during work hours. In this study, we used data from baseline and 18 months follow-up. At baseline, 228 employees received the questionnaire, of which 219 (96%) responded. At 18 months, 152 out of 171 employees (89%) responded. In the current study, we analysed data from the 152 employees with questionnaire data from both points in time. Drop-out from the study was due to employee turnover (*n* = 40), retirements (*n* = 6), relocation to another office (*n* = 4), parental leave (*n* = 4), or sick leave (*n* = 4).

The questionnaire included questions on background characteristics such as age, gender, and variables on health and lifestyle. Space for work material was assessed on a five-level Likert-like scale from “not enough” to “enough”. Availability of daylight and perception of lightning in the office was assessed on a five-level Likert-like scale from “good” to “bad” (Bodin Danielsson 2009). Perceived sit comfort and working posture was assessed on a 4-point scale Likert-like scale ranging from “very good” to “very poor”. At 18 months, a question was added to assess the perceived possibility of adapting workstations based on individual ergonomic needs, using the same 4-point scale. Perceived musculoskeletal symptoms and muscular tension during the past three months were assessed by a 5-point Likert-like scale ranging from “never” to “always”, following Wahlstrom et al. (Wahlström et al. 2004).

#### Statistical analysis

Wilcoxon-matched paired tests were used to test for changes between baseline and 18 months in questionnaire data for all workers and for subgroups. Chi-squared tests were used to test the associations of perceived possibility to adapt ergonomic settings in relation to the level of reported musculoskeletal discomfort. Statistical analyses were performed using SPSS software v.24 (IBM Corp, Armonk, NY, USA), and the significance level was set at α = 0.05.

## Results

### Participants in interviews and baseline characteristics in questionnaires

In total, 77 unique participants (73% women, 27% men) aged 30–63 participated in the interviews. Of the total 77 participants, 62 were employees and 15 were managers. Among the 62 employees, 19 persons participated in more than one interview, and five of the 15 managers participated in more than one interview.

Of the respondents to the questionnaire, 67% were women, and 26% were managers. At baseline, 51% of participants rated their health as “very good” or “excellent”, while 23% reported discomfort in the neck and shoulders either “often” or “all the time” (Table 1).

**Table 1:** Baseline characteristics of participants (*N* = 152)

Demography and work characteristics	Women (%)	Men (%)	All %
*Age groups*			
18–39 years	28.4	26.0	27.6
40–49 years	23.5	30.0	25.7
≥ 50 years	48.0	44.0	46.7
Sex, women			67
Managers	25	28	26
*Hours worked*			
Works full time (40 h/week)	94	98	95
Works part-time (30–39.9 h/week)	6	2	5
*Computer work per workday*			
0–4 h	20	26	22
4–6 h	34	46	38
6–8 h	46	28	40
*Working from home*			
Never	75	74	75
1–4 times per month	23	16	20
Once a week or more	2	10	5
**Health and lifestyle**			
*Self-rated general health*			
Very good or excellent	55	70	60
Good	33	24	30
Fair or bad	12	6	10
*Musculoskeletal discomfort in neck or shoulders*			
Never or seldom	47	60	51
Sometimes	30	20	26
Often or always	25	20	23
*Office type before relocation*			
Personal cell office	71	65	69
Personal workstation in shared room, 2–4 persons	9	12	10
Personal workstation in open landscape	12	12	12
No personal workstation	8	10	9

### Results from interviews and questionnaires

The qualitative results drive the presentation of the data. Where applicable, quantitative findings from questionnaires are interwoven. The qualitative analysis resulted in two themes. The first theme, *reorganising Occupational Health and Safety management*, includes the subthemes *needing new processes and clarification of responsibilities* and *managers navigating the new environment*. The second theme, *one size does not fit all*, includes the subthemes *managing the body in a shared environment* and *challenges of different needs in the workforce.* Themes, subthemes, and categories are presented in Table 2. Further description and clarification of themes and categories are unfolded in the text.

**Table 2:** Themes, subthemes, and categories based on interview data, analysed by qualitative content analysis

Themes	Subthemes	Categories
Reorganising Occupational Health and Safety management	Needing new processes and clarification of responsibilities	Unclear roles, rules, responsibilities, and routines
		A need for restructured safety inspections
		Digital processes are needed for the flex office to be used as intended
	Managers navigating the new environment	New strategies come without guidance
		Imbalance between responsibility and mandates
One size does not fit all	Managing the body in a shared environment	Individual preferences for temperature and lightning
		Time, space, and equipment as barriers to good ergonomics
		Pain and how to handle the new environment
	Challenges of different needs in the workforce	Various needs and preferences depending on work tasks
		Various needs depend on cognitive and physical disabilities

#### Reorganising occupational health and safety management

In this theme, the results highlight how Occupational Health and Safety (OHS) management needed to be reorganised in the AFO, how managers strived to adapt to the new environment, and the challenges these adaptations entailed.

#### Needing new processes and clarification of responsibilities

In the category *unclear roles, rules, responsibilities, and routines*, it was described as unclear who had the overall responsibility for the work environment and who was responsible for the processes involved in making changes to the work environment. Neither managers nor employees knew how OHS management was organised or who was responsible for what. Informants described how the shared environment created the need to handle OHS in new ways, with more complex coordination of stakeholders. As an example of the ambiguities in responsibility, employees described it as unclear how to report deficiencies in the work environment, and it was also unclear who was responsible for solving the problem. Employees found this frustrating and annoying, while others expressed a sense of resignation.

In an environment like this, it becomes even more important to be clear. We cannot act as individual islands anymore. It requires more cooperation, better structure, and professionalism. (Manager, interview 5)

The category *a need for restructured safety inspections* describes the changed prerequisites for carrying out safety inspections. Before relocation, regular safety inspections were held by managers and safety officers in each department. Eighteen months after relocation to the new premises, no safety inspection had been held, and it was still unclear who was responsible for initiating and leading the development of new routines. Informants discussed the challenge in finding new ways to carry out safety inspections, both concerning which stakeholders should be represented, and how to create updated routines and protocols.

“Safety representatives ought to be independent from organisational divisions. The safety representative should represent the whole workplace, regardless of who is sitting where.” (Employee, Focus group 12, 18 months)

In the category *digital solutions are needed for the flex office to be used as intended*, informants explained that the perception of the office’s functionality depended on work tasks and to what extent digitalised work processes were implemented in departments. Employees working with non-digitalised work processes did not perceive the AFO to be supportive. As an example, employees who mainly worked with papers and binders described how they spent more time packing, unpacking, and carrying work materials. Regarding the possibility of storing work material, some perceived the available cabinets to be sufficient, while others found them to be too small. The use of the cabinets seemed to work well for employees who mainly worked in digitalised processes, while employees working with paper and binders were more critical of the cabinets’ storage capacity.

“I work with mail and paper and registration. My desk is full of papers. I cannot keep moving around. I feel rootless, a little stressed, I have to find a place for my papers.” (Employee, Focus group 3, 6 months)

“I usually work with digital processes and have been forced to become even more digital. It works really well.” (Employee, Focus group 6, 6 months)

Questionnaire data showed that 80% of participants reported having enough space for work material at baseline. This decreased to 38% at follow-up (Table 3).

#### Managers navigating the new environment

In the category *new strategies come without guidance*, managers described how the AFO imposed difficulties when it came to handling their subordinates’ work environment issues. After relocation they found it more complicated to help their subordinates with workplace adaptations, as there were no clear guidelines or policies to follow. They tried to support their subordinates and to solve problems as they went along, but due to the shared premises across departmental boundaries, combined with a lack of clear processes and responsibilities, managers perceived that there were limited opportunities to act, leading to frustration among both managers and their subordinates. Managers received some support from human resource departments when they needed help on individual cases, but often they had to come up with solutions themselves.

“My picture is that individuals with special needs are dealt with case-to-case. It’s done in a positive spirit.” (Manager interview 4)

In the category *imbalance between responsibility and mandates*, managers described it as more complicated to take responsibility for their subordinates´ work environment, due to an imbalance between their responsibilities and their mandate to address issues with the workplace environment. They describe situations where they wanted to make adaptations for a subordinate but did not receive any response from top management regarding their inquiries on how they could and should proceed. This led to difficult situations for managers, since they had a responsibility to adapt the work environment according to their subordinates´ needs, but at the same time, they had limited mandates to decide on changes in the shared environment. This imbalance created worse conditions for managers, especially among first-line managers.

### One size does not fit all

This theme explores how the physical work environment, both upsides and challenges, was perceived after relocation to the AFO. Results also describe how the AFO was perceived in relation to a variation of needs.

#### Managing the body in a shared environment

In the subcategory *individual preferences for temperature and lighting*, the indoor environment was reported to be satisfying overall with very good air quality. On the other hand, premises were also described as cold, and informants explained that they often felt cold and used more clothing to keep warm. Temperature differed within the office, and small rooms were perceived as warmer. The office area was perceived as bright, and access to daylight was perceived to be satisfactory, especially on the top floor. Lighting was perceived as unevenly distributed, where some places were perceived as ‘dark’ (desk booths), and participants desired individual desk lights at workstations. The automated lighting was perceived as problematic. It was difficult to understand how to use the light switches, and when turning on a light switch, several areas of the office were affected.

“The indoor air is fantastic here!” (Employee, Focus group 5, 6 months)

“And then these light switches, you can’t find them, and you don’t know which button to press. And then, suddenly, you turn the light off…” (Employee, Go-along interview 5, 6 months)

“…everything works well except that the light is uneven…as I am getting old and need a lot of light.” (Employee, Go-along interview 6 months)

In the questionnaires, access to daylight was rated lower after relocation (*p* < 0.001). The perceptions of lighting quality showed no change after relocation for the whole group (*p* = 0.119), but employees ≥40 years (*p* = 0.031) of age reported worse lighting after relocation, while employees 20–39 years did not (*p* = 0.397) (Table 3).

**Table 3:** Results for space for work material, daylight, and lighting at baseline and 18 months after relocation

		Not enough				Enough	*p*-value
Space for work material, %	Baseline	5.3	4.6	4.6	5.9	79.6	**< 0.001**
	18 months	9.9	18.5	21.2	11.9	38.4	
		Bad				Good	
Availability to daylight, %	Baseline	2.0	2.6	9.9	16.4	69.1	**< 0.001**
	18 months	6.6	8.6	17.2	3.5	37.1	
Perceived lighting, all workers, %	Baseline	3.3	7.9	7.9	24.3	56.6	0.119
	18 months	3.3	8.6	13.9	29.8	44.4	
Perceived lighting depending on age at baseline and 18 months after relocation							
		Bad				Good	
Employees 20–39 years *n* = 42	Baseline	2	7	10	14	67	0.397
	18 months	0	2	5	36	57	
Employees ≥40 years *n* = 110	Baseline	4	8	7	28	53	**0.031**
	18 months	5	11	17	28	39	

Bold indicates statistically significant results between baseline and follow-up

In the category *time, space, and equipment as barriers for good ergonomics*, employees described the ergonomic conditions as being generally good, with great possibilities for variation between sitting, standing, and walking. At the same time, they perceived office ergonomics to be deteriorated and less of a priority than before, and there were some concerns expressed about what the ergonomic consequences might be in the long run. Some thought it was difficult and time-consuming to adjust the chairs and often they choose not to. Instead, they chose to stand up while working, use another chair, or sit at the front edge of the chair. There were also employees who reported that they always took the time to set up their workstation; i.e., due to previous experience with poor ergonomics or problems with musculoskeletal symptoms.

”It takes time to adjust the chair, it’s hard. And you twist and twist and they are hard, and it probably takes 20 minutes. And I must look at the instruction manual every time…” (Employee, Focus group 11, 18 months)

“I always adjust my workstation. I try to be aware of this because I have problems (musculoskeletal symptoms)” (Employee, Focus group 11, 18 months)

“It’s difficult, I can only encourage them to stand up and try to vary their work posture. But from what I hear, many do not have the patience to adjust the workstations. You make some small adjustments, raise and lower the desk a little, move the monitor arm a little, but I’m not sure how much you can really customise it to your own needs”. (Manager interview 6)

Overcrowding and lack of workstations also affected office ergonomics, and informants explained that they did not always find an ergonomically suitable workstation and were forced to work on a sofa, or in the breakroom or other shared spaces, without any possibility of adjusting their chair or table or connecting the laptop to an external monitor. For example, sitting on the sofa working on a laptop led to forward bending of the neck. Other obstacles mentioned were that the technical settings on the computer could be spoiled when switching workstations if the computer had not been docked. This made employees remain at the same workstation instead of switching.

“… you only use the sofas if there’s no other workstation available.” (Employee, Go-along interview 2, 6 months)

Ratings for sit comfort and work posture decreased significantly after relocation. This decrease seemed to be driven by older employees, persons with neck- and shoulder symptoms at baseline, and women (Table 4 and Supplementary Table 2).

**Table 4:** P-values for perceived changes in sitting comfort, working posture, and musculoskeletal symptoms at baseline compared to 18 months after relocation in the whole group and different subgroups

	All *N* = 152	20–39 years *n* = 42	≥40 years *N* = 108	Neck/shoulder pain at baseline^1^ *n* = 35	Not neck/shoulder pain at baseline^2^ *n* = 115	Men *n* = 50	Women *n* = 101
Sitting comfort	**> 0.001**	0.580	**> 0.001**	**> 0.001**	**0.023**	0.104	**> 0.001**
Working posture	**> 0.001**	**0.042**	**> 0.001**	**0.003**	**0.012**	0.218	**> 0.001**
Neck and shoulder pain	**0.007**	**0.005**	0.135	0.151	**> 0.001**	0.139	**0.025**
Back, %	0.081	0.059	0.385	0.588	0.077	0.961	**0.027**
Hip, knee or feet, %	0.602	0.221	0.987	0.986	0.557	0.824	0.477
Headache, %	0.216	**0.005**	0.603	0.330	0.383	0.451	0.319
Muscle tension, %	0.789	0.480	0.927	0.815	0.641	0.780	0.624

^1^ Never, seldom, or sometimes pain in neck and shoulder at baseline

^2^ Often or always pain in neck and shoulder at baseline

Bold indicates statistically significant results

In the category, *pain and how to handle the new environment*, some employees report that they had more problems with headaches and back, neck, and shoulder discomfort or pain after relocation. They believed that the increase was due to difficulties adjusting the workstation. Their symptoms were triggered when the current workstation was not adjusted correctly, or when working mainly on a laptop. Informants also mentioned that they tended to sit closer to the laptop screen when doing high-concentration work.

Employees report different strategies for managing discomfort and pain in the new office. Some used the treadmills in the office to relieve back pain, while others appreciated the daily digital prompts for exercise programs. Other informants, however, felt exposed when using the exercise program in the open office environment. To achieve good ergonomic conditions and avoid discomfort and pain, individual responsibility and self-care were mentioned as being more important in the new office environment. To achieve a sustainable situation, some employees claimed a personal room or described leaving their things at the same workstation to ‘keep’ the workstation for the whole day.

“I’ve got more problems with my neck, shoulders, back, and everywhere. I’m sitting badly. I’ve had problems before and then my chair and table were adjusted to my needs. Now it’s hard to adjust all the settings every morning…” (Employee, Focus group 6, 6 months)

“…instead, I have become more selfish and pick a quiet workplace or a room of my own as quickly as I can, and don’t bother to change during the day.” (Employee, Focus group 11, 18 months)

The questionnaires showed a significant increase in reported neck and shoulder discomfort between baseline and 18 months (*p* < 0.007) in the whole group (Supplementary Table 3). Subgroup analysis revealed that the increase in reported neck and shoulder pain seemed to be driven by younger employees; employees that reported to never, seldom, or sometimes experience neck and shoulder symptoms at baseline; and by women (Table 4). Those who reported musculoskeletal discomfort and muscular tension at 18 months after relocation also reported fewer possibilities to adjust their workstation ergonomically compared to those who did not (Supplementary Table 4).

#### Challenges of different needs in the workforce

In the category, *various needs and preferences depending on work tasks*, employees described that the type of work tasks affected the extent to which they took advantage of the new office. Employees who mainly worked on stationary screen-based work found no incentives to change workplaces during the day. In contrast, those attending many meetings needed to find new workstations between meetings, which could be a challenge, due to crowdedness. Employees with high-concentration work tasks complained that they spent time finding secluded rooms or a quiet place so they could concentrate. The possibility of arriving early at the office to secure a suitable workstation was described as a problem, as it was not possible for everyone to arrive early.

In the category *various needs depending on cognitive and physical disabilities*, there was a critique that individual adaptation needs were not mapped and considered sufficiently before relocation. It was emphasised that this should have been considered already during the planning phase, to be able to account for and accommodate the premises for various needs. Furthermore, it was perceived as difficult—for both employees and managers—to decide when an individual adaptation was needed, and in what cases the individual could handle their individual adaptation in the shared office environment without a targeted adaptation.

Employees perceived it to be more complicated to obtain individual ergonomic aids in the new office environment after the relocation. The reasons for this could be that they did not receive any response from management, or that they simply did not know how to go about requesting an adaptation. Some also felt hesitant about expressing their needs due to concerns about feeling singled out or stigmatised; for example, if a personal room was desired. Furthermore, it was described as easier to ask for equipment and support for physical problems than for psychological issues. Another barrier that was mentioned in the interviews was that it could be burdensome to carry around physical aids (e.g., chairs, armrests, mice, etc.) and that it took time to install them.

“The basic mistake is the insufficient mapping of needs (depending on various work tasks)…The needs vary. I think that a mix of activity-based and cell offices had been better. But the perfect office does not exist…” (Employee, Focus group 10, 18 months)

”It has to be equal for everybody, but we are all different.” (Employee, Focus group 7, 6 months)

## Discussion

The aim of this study was to investigate the physical work environment in an organisation after relocation from a cell office to an AFO using a mixed-methods design.

We found a need for clarified roles and updated processes to handle OHS management. We also found that managers perceived it to be more complicated to help their subordinates with work environmental issues, indicating a need to clarify how managers should handle individual accommodations for employees. Our results regarding OHS management may have been reinforced by the shortcomings of the office design, with crowdedness and perceived challenges finding suitable workstations for concentrated work. The crowdedness itself is an indicator of the lack of holistic OHS management, as no one had the overarching responsibility for the number of employees located in the building. Babapour et al. (2019) also found it important to establish continuous, proactive, and inclusive processes for customising AFOs after relocation to address work environmental problems, and highlight the need to pay close attention to OHS managerial processes in organisations once AFOs are implemented. Our results also illuminate the importance of establishing new ways of organising OHS management. In the post-pandemic situation, when hybrid work is more common, this complexity might increase further. Organisations need to find ways to manage OHS to avoid the possible negative aspects of working from home and blurry lines in work environment responsibility.

Both qualitative and quantitative data revealed that ergonomics and musculoskeletal symptoms after relocation to ABW were negatively affected. Quantitative data suggest that this was driven by young female employees with low or no symptoms at baseline. The qualitative data revealed that desk sharing in AFOs could challenge ergonomics due to the time it takes to set up the workstation, the knowledge of how to adapt the workstation, and the availability of the right space and equipment. In line with our results, Berthelsen et al. (2018) also reported decreased sitting comfort and working postures after relocation to a flex office, and another Swedish study (Babapour Chafi and Rolfö 2019) found that limited time and knowledge could be a barrier to proper ergonomics in flex offices. A recent cross-sectional study found no differences in pain prevalence in most body areas when comparing workers in AFOs and cell offices, but workers in AFOs reported significantly more pain in the right hand, wrist, and fingers, and authors discuss that this might be explained by the daily use of different workspaces and work equipment (Argus and Paasuke 2021). In line with our results, Kim et al. ( 2016) found the shortage of available desks on busy days and the need to provide easy-to-adjust chairs and desks to be important from an ergonomic perspective in AFOs. Contrasting our results, Meijer et al. ( 2009) found decreased musculoskeletal symptoms after relocation from shared cell offices to an innovative office with desk sharing. In their study, the furniture before relocation was not adjustable and employees were not informed about how to properly adjust them, while more ergonomic furniture was purchased for the new office. These differences might explain the different findings of Meijer et al. (2009). Robertson et al. (2008) also found positive effects on the prevalence of musculoskeletal discomfort after relocation to AFOs, especially in the group of workers who participated in the development of the workspace design and in a two-hour ergonomic training session. Therefore, it is likely to be of the utmost importance that organisations implementing flexible office environments make sure their employees have good knowledge of why and how to set up their workplace (Sanaeinasab et al. 2018). In our study, employees were offered a non-mandatory ergonomic training session and an individual ergonomic consultation if they wanted them. Unfortunately, we have no data on the attendance rate for the training session, but they were rarely mentioned in the interviews, which leads us to believe that attendance was low. The implemented intervention during the study period, aiming to decrease sitting and increase physical activity, showed no changes in sitting time or in the temporal patterns of sitting at work, but there was an increase in time spent walking and in moderate-to-vigorous physical activity in the group relocating to the flex office (Wahlström et al. 2019). Thus, the reported changes in perceived sit comfort, working posture, and musculoskeletal pain were likely unaffected by any changes in the temporal patterns of sitting or standing. If any, the effect on ergonomics from the intervention should be a mitigating effect on physical activity, as it is associated with fewer musculoskeletal symptoms.

We also found that older employees reported the lighting to be less satisfactory after relocation. These results illuminate the need to take various needs into account when designing visual ergonomics in the office (Peiyi 2014; Erdinest et al. 2021). The choice of lighting design and control strategies for lighting depends on different factors, such as the number of workers who use the surface, the visual tasks to be performed, and the visual abilities of occupants; e.g., age or other conditions affecting vision (Osterhaus et al. 2015). Before relocation, all cell offices were located along the façade, with direct access to windows and daylight. After relocation, the full depth of the building was utilised for workstations, which led to less availability of daylight and limited access to windows. Based on information in the interviews, we found that the reasons for this deterioration could also be due to perceived difficulties in handling the light switches, which might have caused insufficient and unevenly distributed indirect lighting. Visual comfort has been shown to impact productivity, health, and overall comfort among office workers (Peiyi 2014; Robertson et al. 2016; Candido et al. 2019). Our results exemplify the importance of providing good visual ergonomics via a lighting design with sufficient indirect lighting and the availability for direct lighting where needed, especially for older workers (Osterhaus et al. 2015). To our knowledge, there are no previous longitudinal studies investigating the effects of visual comfort in AFOs, and further studies are needed.

Furthermore, we found that the AFO did not match the prerequisites with the variety of work tasks performed within the organisation. This affected the extent to which employees perceived how they could benefit from the AFO. Employees in the AFO with concentration-demanding work tasks complained about the time spent searching for a suitable secluded workstation. This could potentially affect productivity, since work tasks and a high degree of workspace switching have been shown to be positively associated with productivity and well-being in AFOs (Seddigh et al. 2014; Haapakangas et al. 2019). Previous results from the AOD study (Öhrn et al. 2021) also indicate that flexible and interactive work tasks are more appropriate in AFOs compared to individual high-concentration tasks, and a recent Finnish study (Haapakangas et al. 2022) found that both task-related (related to interactive needs, concentration demands, and managerial position) and person-related factors (age, sex, work ability, and satisfaction with ergonomics) influenced the use of the office and the perception of the person–environmental fit of the AFOs.

Our results highlight the importance of planning and accounting for workers’ individual accommodation needs when relocating to AFOs. In the current study, both managers and employees described various challenges in handling individual workplace adaptations after relocation. The finding that employees with musculoskeletal discomfort reported fewer possibilities to adjust their workstations ergonomically in the AFO is important, since workplace adaptations and workplace support seem to be of great importance in allowing people with disabilities to participate in the workforce (Anand and Sevak 2017). Research on the need and use of work accommodation in terms of adaptations and adjustments to work support is limited, especially in relation to different office types, and to our knowledge, there are no studies regarding workplace adaptations in AFOs. However, studies have shown that the most-needed workplace adaptations are modifications to and accommodations in the work environment and work tasks; for example, scheduling flexibility and accessible workstations (Jetha et al. 2021). A recent paper highlights the need to offer an inclusive environment for all employees and describes the development of a post-occupancy method to evaluate inclusion, diversity, equity, and accessibility in the built environment (Zallio and Clarkson 2022). When implementing AFOs, we suggest that a thorough analysis of work tasks and individual needs should be performed in the planning phase, to be able to design the premises in the best possible way for all employees.

### Methodological considerations

The strengths of this study are its long-term follow-up, the rich qualitative data, and the high response rates on questionnaires providing complementary data and a possibility for a deeper understanding of the sociocultural context and the real-world environment for workers and managers (Gjerland et al. 2019). When using mixed methods, the researcher will end up with a more comprehensive and complete understanding of the problem and potential solutions (Vedel et al. 2018). The qualitative data offers information on how participants interpret and explain their thinking and behaviours when handling, i.e., workplace ergonomics, and thereby offers clues as to possible preventive actions. The use of qualitative content analysis is suitable for studies with an exploratory purpose and repeated discussions among the authors, and when combined with presentations and reflections from other researchers involved in the AOD study it also strengthens the trustworthiness of the study (Priest et al. 2002). This offered the possibility to identify how the combination of a poor office design combined with management failure to prepare employees for the transfer to AFOs seemed to lead to a deterioration in employees’ ergonomic situations and increased musculoskeletal symptoms after relocation. Relocating to an activity-based office does not just involve a change in office design, but implies a whole new way of working, and the individual adaptation to the organisational change is likely to differ between employees. Therefore, a strength in our study is the long-term follow up which has given employees the opportunity to adapt to the new work situation.

Weaknesses that merit mentioning are that the study was conducted within a single organisation in Sweden and that the current study includes no formal control group. As organisations differ substantially in terms of work environment legislation, OHS management, and how the AFO implementation process is performed, the transferability of our findings might be limited. In this study, simultaneous activities and processes—such as the programme for promoting physical activity and the sizing issues, leading to crowdedness—could affect the results. By providing detailed descriptions of the context, study design, participants, analysis process, and by using quotations from study participants, we have facilitated the reader’s ability to interpret the transferability of the results (Nielsen and Randall 2013). In the interviews, the informants were men and women, and managers and non-managers of various ages, which could be assumed to be a representative sample. However, the representation of informants could be biased, as employees who have strong opinions, both positive and negative, might be more prone to sign up for participation when invited to interview. However, data from the interviews showed a large variation, which suggested that the results capture a wide range of experiences, opinions, and feelings that both workers and managers had in relation to the AFO. In sum, we believe that the results could be applicable not only to organisations in Nordic countries but also in a wider international context.

In the future, hybrid work is expected to increase (Hopkins and Bardoel 2023), and our study brings forward important aspects of work environment issues in relation to flexible work environments. Ergonomic concerns as a risk of remote work have been highlighted before, and the International Labor Organisation points out that training and awareness initiatives for both employees and managers are needed to handle the potential risks of remote work (ILO 2017; Chafi et al. 2021). Even though the legislation might differ between different national contexts, OHS management is likely to become more individualised. We argue that when working in AFOs or remotely, there is a shift towards increased individual responsibility for occupational health risks, which means that employees need to be equipped with both knowledge and adequate tools.

### Practical implications

To create a sustainable work environment and a healthy workforce, employers must take preventive measures when designing the office. Further, to settle OHS issues, organisations must engage in continuous dialogue between concerned stakeholders, with the aim of clarifying areas of responsibility and processes related to the flexible work environment. A practical implication of our study is also that ergonomics still matters, and we suggest that ergonomic training for all employees should be mandatory if working in an AFO with desk sharing. Our results also highlight the importance of equipping the office with furniture and equipment that are easy to use and robust enough to withstand repeated changes in settings.

## Conclusions

The implementation of AFOs based on activity-based work is a complex process. Our findings indicate a need to reorganise and clarify roles and processes for OHS management when implementing an AFO. We also found office ergonomics and musculoskeletal symptoms to be negatively affected after relocation. These effects were influenced by a combination of factors, such as difficulties adjusting the workstations, a lack of suitable workstation due to crowdedness, age, sex, and a variety of needs relevant to different groups as well as individuals. Long-term health effects after implementing AFOs are understudied, and there is an urgent need for more longitudinal studies in larger samples to increase knowledge and support organisations and policymakers in successfully handling the complex process of implementing AFOs.

### Electronic supplementary material

Below is the link to the electronic supplementary material.

Supplementary Material 1

## Data Availability

The data that support the findings of this study are not openly available due to reasons of sensitivity and are available from the corresponding author only upon reasonable request. Data are located in controlled access data storage at Umeå University.
