# Supplementary material for: Physical work environment in an activity-based flex office: a longitudinal case study
Source: Int Arch Occup Environ Health. 2024 May 17;97(6):661–74. doi: 10.1007/s00420-024-02073-z (PMC11245412; doi:10.1007/s00420-024-02073-z)
Supplement: Supplementary file 1 — Supplementary Material 1 [file 420_2024_2073_MOESM1_ESM.docx]

## Supplementary tables

| **Supplementary Table 1.** Organisational and architectural features in the activity-based flex-office (AFO) at 18 months follow-up^*^ | |
| --- | --- |
| Number of employees |  |
| Number of employees scheduled to work in the office | 270 |
| Number of employees at 18 months follow-up | 315 |
| **Organizational conditions** |  |
| Clean desk policy^a^ | Applied |
| Personal workstations^b^, % | 2% |
| Shared workstations | Yes |
| Policy for zones^c^ | No application of zones |
| ICT solutions^d^ | Wireless network  Laptops and USB docking stations  Skype for business  Air media (blue tooth)  Mobile phones  VPS tunnel (CISCO) |
| **General architectural features** |  |
| Total area (m^2^)^e^ | 4805 |
| Area per person (m^2^)^f^ | 15 |
| Number of floors | 2 (+1 reception floor) |
| **Workstations** |  |
| Total number of workstations (des, chair, computer, screen), n | 160 |
| Workstations in open plan offices, n | 116 |
| Secluded rooms (1–2 persons), n | 44 |
| **Supportive areas** |  |
| Touch down seats, n | 46 |
| Touch Down tables, n | 13 |
| Number of seats at group tables in open plan offices, n | 30 |
| Number of seats in small meeting rooms (2–6 persons), n | 52 |
| Number of seats in large meeting rooms (7–25 persons), n | 56 |
| **Lounges and break-out spaces** |  |
| Sofas and lounge chairs, number of seats  Break out spaces, number of seats  Number of standing height tables | 160  193  17 |
| **Total number of seats in the office** | 697 |

^*^Modified from Öhrn et al 2021.

^a^Clean desk policy = when leaving the workstation all personal belongings must be removed

^b^Personal work station = a work station assigned for an individual employee

^c^Policies for zones: Application of rules for different sound levels and behaviour in different areas of the office.

^d^ICT-solutions = Information and Communication Technology

^e^Total area (m^2^) = measured from printed drawings of the office area included in this study using gross external area (GEA), according to British governmental standards (https://www.rics.org/globalassets/rics-website/media/upholding-professional-standards/sector-standards/valuation/code-of-measuring-practice-6th-edition-rics.pdf ).

^f^Area per person (m^2^): Calculated as the ratio from total area and actual number of employees at time of study.

| **Supplementary Table 2.** Perceived sitting comfort and working posture at baseline and 18 months after relocation. Bold indicates statistically significant results between baseline and follow-up. | | | | | | |
| --- | --- | --- | --- | --- | --- | --- |
|  |  | Very bad |  |  | Very good | P-value |
| Sitting comfort, % | Baseline | 0 | 5.9 | 49.3 | 44.7 | **<0.001** |
|  | 18 months | 5.9 | 15.1 | 50.0 | 28.9 |  |
| Working posture, % | Baseline | 1 | 2 | 47.4 | 50.0 | **<0.001** |
|  | 18 months | 3.4 | 14.8 | 47.7 | 34.2 |  |

| **Supplementary Table 3.** Musculoskeletal symptoms at baseline and 18 months after relocation. Bold indicates statistically significant results. | | | | | | | |
| --- | --- | --- | --- | --- | --- | --- | --- |
| All workers |  | Never | Seldom | Some-  times | Often | Always | P-value |
| Neck and shoulder, %  n =150 | Baseline | 25.7 | 25.0 | 26.3 | 11.8 | 11.2 | **0.007** |
|  | 18 months | 19.3 | 22.7 | 28.7 | 15.3 | 14.0 |  |
| Back, %  n =150 | Baseline | 34.2 | 31.6 | 15.8 | 11.2 | 7.2 | 0.081 |
|  | 18 months | 29.1 | 29.8 | 19.9 | 10.6 | 10.6 |  |
| Hip, knee or feet, %  n =150 | Baseline | 38.2 | 26.3 | 12.5 | 13.2 | 9.9 | 0.602 |
|  | 18 months | 38.9 | 24.2 | 12.8 | 10.7 | 13.4 |  |
| Headache, %  n =150 | Baseline | 29.6 | 35.5 | 22.4 | 11.8 | 0.7 | 0.216 |
|  | 18 months | 26.7 | 35.3 | 22.0 | 12.7 | 3.3 |  |
| Muscle tension, %  n =150 | Baseline | 20.5 | 24.5 | 31.1 | 17.2 | 6.6 | 0.789 |
|  | 18 months | 19.3 | 28.0 | 25.3 | 20.7 | 6.7 |  |

| **Supplementary table 4.** Perceived possibilities to adjust the workstation ergonomically at 18 months among employees with and without musculoskeletal symptoms in different body regions.  Bold indicates statistically significant results. | | | | | | |
| --- | --- | --- | --- | --- | --- | --- |
|  |  | Very bad | Quite bad | Quite good | Very good | P-value |
| Neck and shoulder pain, %, n =150 | Never, seldom or sometimes | 2.7 | 9.3 | 32 | 26.7 | **<0.001** |
|  | Often or always | 5.3 | 9.3 | 10 | 4.7 |  |
| Back pain, %  n =151 | Never, seldom or sometimes | 3.9 | 13.2 | 35.8 | 25.8 | **0.019** |
|  | Often or always back pain | 3.9 | 6 | 6 | 5.3 |  |
| Pain in hips, knee or feet, %  n =149 | Never, seldom or sometimes | 4.7 | 14.8 | 31.5 | 24.8 | <0.469 |
|  | Often or always | 3.3 | 4.7 | 10 | 6 |  |
| Headache, %  n =150 | Never, seldom or sometimes | 4.6 | 15.3 | 35.3 | 28.7 | **0.038** |
|  | Often or always | 3.3 | 4.0 | 6.0 | 2.7 |  |
| Muscle tension, %  n =150 | Never, seldom or sometimes | 2.7 | 8.7 | 34.0 | 27.3 | **<0.001** |
|  | Often or always | 5.3 | 10.7 | 8.0 | 3.3 |  |
